# Misdiagnosis of Herlyn–Werner–Wunderlich syndrome combined with pelvic mesothelial cyst: a case report

**DOI:** 10.3389/fmed.2025.1513725

**Published:** 2025-02-12

**Authors:** Jinrong Li, Qiong Duan, Na Long, Fengming Ji, Yu Hang, Zhen Yang, Yucheng Xie, Chenghao Zhanghuang, Bing Yan

**Affiliations:** ^1^Department of Urology, Kunming Children’s Hospital, Yunnan Province Clinical Research Center for Children’s Health and Disease, Kunming, China; ^2^Special Ward, Kunming Children’s Hospital, Kunming, China; ^3^Department of Oncology, Yunnan Children Solid Tumor Treatment Center, Kunming Children’s Hospital, Kunming, China; ^4^Department of Pathology, Kunming Children’s Hospital, Kunming, China; ^5^Yunnan Key Laboratory of Children’s Major Disease Research, Yunnan Clinical Medical Center for Pediatric Diseases, Kunming Children’s Hospital, Kunming, China

**Keywords:** Herlyn–Werner–Wunderlich syndrome, mesothelial cyst, diagnosis, treatment, case report

## Abstract

Herlyn–Werner–Wunderlich syndrome (HWWS) combined with pelvic mesothelial cyst is a rare condition that can be easily misdiagnosed. Our center presents a case study of this disease to provide clinicians with valuable insights for accurate diagnosis and treatment. A 12-year-old girl initially received a misdiagnosis of left lower abdominal ectopic kidney hydronephrosis based on B-ultrasound and renography due to intermittent abdominal pain. However, surgical exploration revealed the correct diagnosis of HWWS with pelvic mesothelial cyst. Postoperative pathology confirmed the presence of a mesothelial cyst in the pelvis. Once the diagnosis was confirmed, the patient was transferred to an adult gynaecology department for further treatment. Two months later, during follow-up via telephone, it was reported that the patient had successfully recovered after undergoing transvaginal oblique septum resection. This case report provides detailed information on the diagnostic process, treatment procedures, and examination results, serving as a valuable reference for clinical practitioners dealing with HWWS combined with pelvic mesothelial cysts while reducing the risk of misdiagnosis and mistreatment.

## Background

The Herlyn–Werner–Wunderlich syndrome (HWWS) represents a rare congenital malformation of the urogenital system, defined by vaginal atresia that may be complete or incomplete due to the presence of an oblique septum. This syndrome is commonly associated with a uterus didelphys (alternatively, a septate or bicornuate uterus), double cervix, and an oblique vaginal septum, and frequently co-occurs with urinary system anomalies on the affected side. The most common associated anomaly is renal agenesis, although other renal conditions such as multicystic dysplastic kidney, duplicated kidney, ectopic fused kidney, or ectopic ureter may also be observed. First described by Gasser et al. (1) the syndrome’s current appellation is based on two subsequent reports. Herlyn and Werner (2) described the co-occurrence of a Gartner’s duct cyst, ipsilateral renal hypoplasia, and a uterus didelphys, which they named Herlyn–Werner syndrome. Wunderlich (3) reported an association between right renal hypoplasia and a uterus didelphys with a simple vagina, along with an isolated hematometra of the cervix, resulting in the syndrome being designated as HWWS.

HWWS has an incidence rate of about 0.1 to 3.8% (4). Although the exact pathogenesis of HWWS is unclear, it is believed to be linked to anomalies in the development of the Müllerian and Wolffian ducts (5). HWWS is typically accompanied by other medical issues, with endometriosis being the most frequently encountered (6). Extensive literature reviews, both domestically and internationally, have not uncovered any cases of HWWS combined with pelvic mesothelial cysts. We present the case of a 12-year-old female patient who sought medical attention due to a pelvic mass and abdominal pain. Preliminary assessments using ultrasonography and renal scintigraphy led to a misdiagnosis of ectopic kidney with hydronephrosis. Additional findings included renal dysplasia and left ureteral dilation. Upon laparoscopic investigation and postoperative histopathological confirmation, the patient was diagnosed with HWWS along with a pelvic mesothelial cyst. She was subsequently transferred to a gynecological hospital for further surgical management.

## Case report

A 12-year-old female patient presented to our hospital with intermittent abdominal pain 1 month ago. An outpatient ultrasound of the urinary system revealed a mass in the left lower abdomen, measuring approximately 7.8 × 6.8 × 5.2 cm, with a wall thickness of about 0.51 cm. The mass was oval-shaped, with clear boundaries and an anechoic interior, exhibiting poor acoustic transmission. Color Doppler Flow Imaging (CDFI) showed no blood flow signals within the anechoic area, but punctate blood flow signals were observed on the wall of the mass. The mass appeared to be connected to the ureter, with the left ureter being markedly dilated, with a maximum internal diameter of about 3.5 cm, and a tortuous course, also showing poor acoustic transmission.

**Figure 1:**
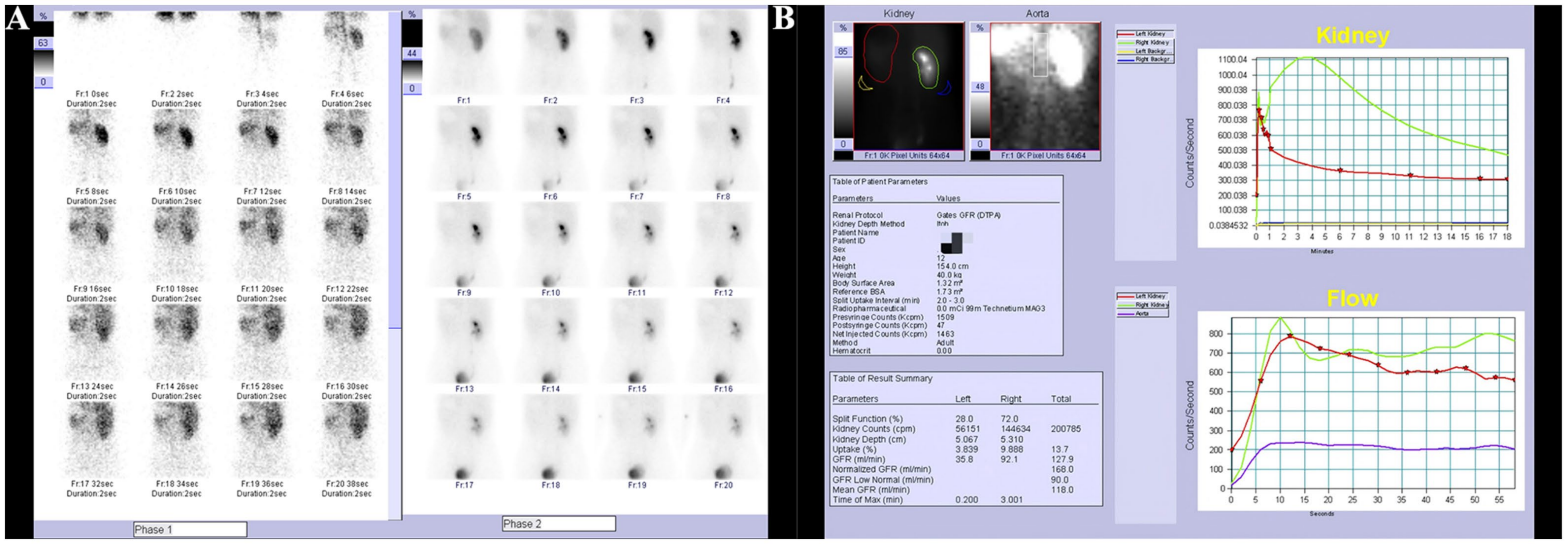
Renal scan results. **(A)** Slight enlargement of the left kidney with hydronephrosis, normal blood flow perfusion. **(B)** Mild to moderate impairment of glomerular filtration function, slow upper urinary tract drainage.

Ultrasonography of the uterus and bilateral adnexa revealed no abnormalities. After administration of oral analgesics, the patient’s pain was alleviated, and they were subsequently referred to an external hospital for renal scintigraphy. The renal scintigraphy performed at the external hospital indicated a glomerular filtration rate (GFR) of 35.8 mL/min for the left kidney and 92.1 mL/min for the right kidney. The left kidney showed slight enlargement with signs of hydronephrosis, while blood flow perfusion was normal. Renal glomerular filtration function was mildly to moderately impaired, and the upper urinary tract drainage was slow (Figures 1A,B).

Physical examination revealed normal development of secondary sexual characteristics and normal external genitalia, with an intact hymen. The abdomen was soft, with mild tenderness in the left lower quadrant and no palpable masses. There was no percussion tenderness over the renal areas, and no significant tenderness at the ureteral points. During the course of the illness, the patient experienced intermittent pain in the left lower abdomen, which was alleviated by oral analgesics. The patient did not report urinary frequency, urgency, dysuria, or difficulty in urination. There were no signs of fever, nausea, vomiting, edema, abdominal bloating, or diarrhea. The patient’s mental status, appetite, and sleep were normal, and there was no significant change in body weight.

**Figure 2:**
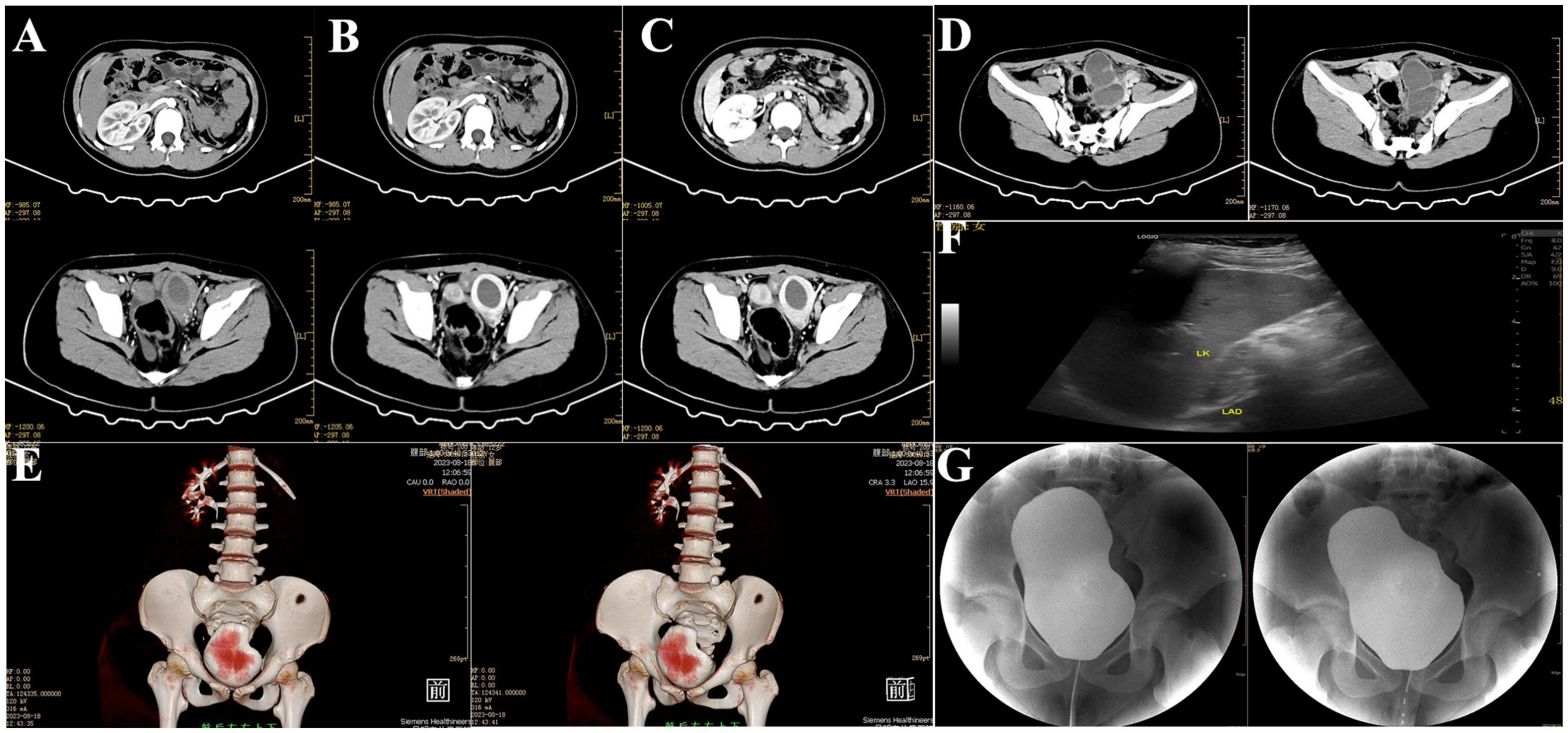
Preoperative imaging findings. **(A–C)** Contrast-enhanced cross-sectional CT of the abdomen and pelvis revealed bilateral uterine malformations with a left-sided hematoma. **(D)** Enhanced CT image of the left pelvic mesothelial cyst. **(E)** Three-dimensional reconstruction of abdominal and pelvic CT. **(F)** Preoperative urinary system and gynecological ultrasound. **(G)** Preoperative retrograde urography.

Outpatient diagnosis: “(1) left ectopic kidney; (2) left ectopic kidney hydronephrosis; (3) left renal colic; (4) left renal hypoplasia; (5) left ureteral dilation.” The patient was admitted for further management. The patient denied any sexual activity and reported menarche at age 11 with generally regular menstrual cycles. However, the menstrual blood volume was irregular, with a dark red color. Over the past 2 months, the patient experienced increased abdominal pain during menstruation. Upon admission, a comprehensive enhanced CT scan of the abdomen was performed, which revealed: (1) an abnormal density focus in the left pelvic area, suggesting further MRI examination to rule out the presence of a uterus didelphys or other anomalies; (2) a vacant left renal area with a multicystic, low-density shadow in the left pelvic region. The lesion was multicystic, low-density, and non-enhancing, with a CT value of 12 HU and a maximum dimension of 5.5 cm × 4.6 cm; (3) a small amount of pelvic effusion (Figures 2A–E).

Ultrasonography of the urinary system revealed: (1) a thick-walled cystic mass in the left lower abdomen, suggestive of a tortuous ureter; (2) abnormal shape of the left kidney with small size and mild hydronephrosis, indicative of renal hypoplasia (Figure 2F). Retrograde urography showed no reflux in the bilateral ureters (Figure 2G). Clinical diagnosis: (1) pelvic mass of undetermined etiology, suspected to be hydronephrosis of an ectopic kidney with ureteral elongation; (2) uterus didelphys cannot be ruled out.

**Figure 3:**
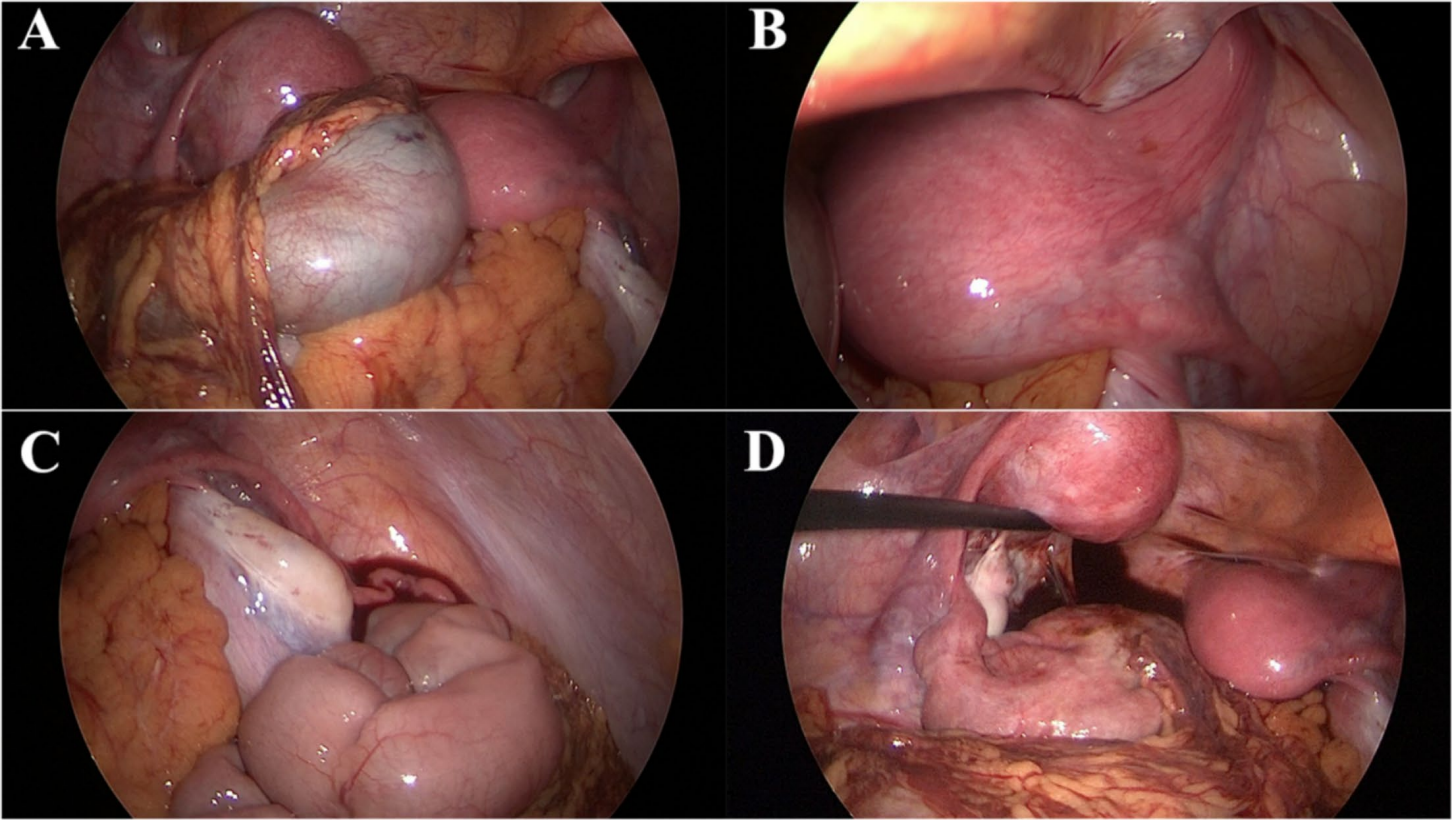
Laparoscopic surgical findings. **(A)** Uterus didelphys with each horn connected to the corresponding ovary and fallopian tube. **(B)** Right uterus. **(C)** Smooth cystic mass adjacent to the left fallopian tube with a ruptured base and chronic inflammatory adhesions of surrounding tissues. **(D)** Squeezing the mass results in the outflow of old, pale bloody fluid from the rupture site.

**Figure 4:**
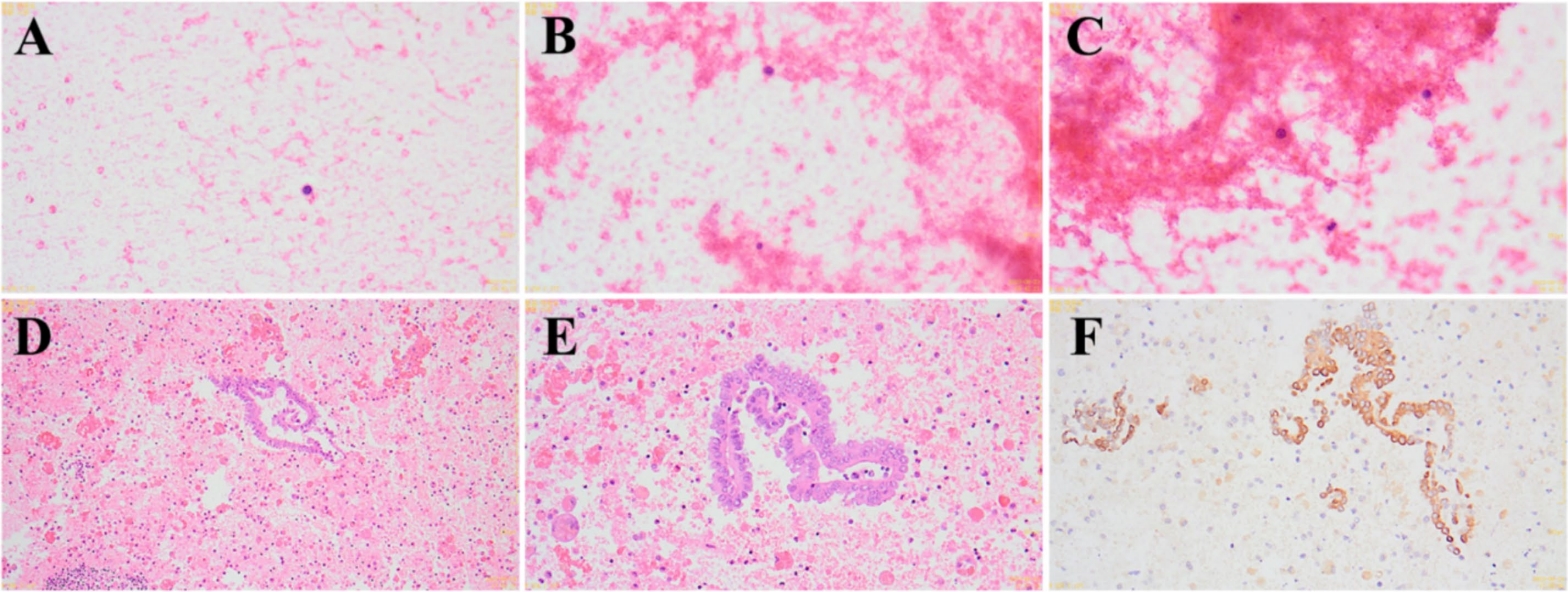
Intraoperative and postoperative pathological findings. **(A–C)** Intraoperative pathological findings show a few lymphocytes and scattered histiocytes in a bloody background (**A**: 400×; **B**: 400×; **C**: 400×). **(D–F)** Postoperative immunohistochemistry results suggest a mesothelial cyst (**D**: 100×; **E**: 400×; **F**: 400×).

After admission, the patient underwent comprehensive preoperative examinations to rule out any contraindications to surgery and subsequently underwent a laparoscopic exploration under general anesthesia. Laparoscopic examination revealed a uterus didelphys, each connected to the corresponding ovary and fallopian tube (Figure 3A). The left uterus was hypoplastic, measuring approximately 3.5 cm × 3.0 cm × 2.5 cm, while the right uterus measured approximately 5.5 cm × 4.6 cm × 4.0 cm. A cystic mass, approximately 5.0 cm × 3.0 cm × 2.6 cm in size, was observed adjacent to the left fallopian tube. The mass had a smooth surface and a ruptured base, with surrounding tissues showing chronic inflammatory adhesions. Squeezing the mass resulted in the outflow of old, pale bloody fluid from the rupture site, suggesting a possible diagnosis of endometriosis with hemorrhage during the operation (Figures 3B–D). A portion of the fluid was sent for pathological examination, and the intraoperative pathological results showed a few lymphocytes and scattered histiocytes in a bloody background, with no tumor cells or fungi detected (Figures 4A–C). A thorough exploration of the abdominal cavity revealed no left ureter or renal tissue. Intraoperative diagnoses included: (1) vaginal septum syndrome; (2) pelvic mass of undetermined etiology (suspected endometriosis with hemorrhage or other possibilities).

After clarifying the diagnosis, it was determined that our hospital lacks the relevant gynecological diagnostic and treatment experience, and our department does not have the surgical experience for this condition. During the operation, we consulted with experts from a higher-level hospital, who advised halting the surgery and transferring the patient for elective surgery at a higher-level facility. The situation was explained to the family, and the surgery was terminated. Postoperative analysis of the old, pale bloody fluid revealed a pink-stained inflammatory background with segmental epithelial cells, some showing papillary hyperplasia, and lymphocytic hemorrhagic cysts. Immunohistochemical staining results were as follows: CD20(−), CD3(−), CD163(−), MC(+), CR(+), CK8/18(+), Ki-67(5%+), PR(partially+), a-inhibin(−), CK5/6(+). These findings support the diagnosis of a mesothelial cyst (Figures 4D–F). After discharge, the patient was transferred to the gynecology department of an adult hospital for further treatment. Follow-up revealed that, due to the absence of sexual activity, the patient underwent a non-invasive hymenal hysteroscopic vaginal septum resection, and the recovery has been good.

## Discussion

HWWS combined with pelvic mesothelial cysts is a rare clinical condition. The etiology of HWWS remains unclear. It is currently believed that during the development of the urinary and reproductive systems in normal females, certain factors may lead to the termination of development of one side of the mesonephric duct, which in turn halts the development of the corresponding Müllerian duct. However, the development of the other side of the mesonephric and Müllerian ducts remains unaffected, resulting in a unilateral urinary and reproductive system and causing asymmetrical genital tract malformations combined with urinary system malformations (7, 8). Symptoms in patients with HWWS are primarily related to the inability to drain blood and pus from the vaginal cavity behind the septum, such as menstrual pain, prolonged menstruation, or abnormal vaginal bleeding outside of the menstrual period (9, 10). Gynecological ultrasonography is the preferred method for diagnosing HWWS. However, gynecological ultrasound is not suitable for young females without a history of sexual activity, and if the examining physician is not familiar with HWWS, misdiagnosis and missed diagnosis can easily occur (11). Pelvic MRI can clearly display blood and pus in the uterine cavity and vagina, and is useful for understanding the level of vaginal obstruction and the structure of the uterus and cervix. If necessary, physiological saline can be injected into the vagina to make the MRI imaging clearer. Therefore, pelvic MRI is considered one the most important auxiliary imaging examination for diagnosing HWWS (12). In this case, MRI was not performed, and thus a correct preoperative diagnosis was not achieved. CT is not a necessary diagnostic tool for HWWS, especially for underdeveloped young patients.

Mesothelial cysts are benign lesions originating from mesothelial cells that cover the serosal cavities. These cysts are typically small, usually less than 5 cm in diameter, and are often solitary and unilocular, although they can occasionally consist of 2–3 locules. The cyst walls are thin, and the inner lining is composed of well-differentiated flat mesothelium without significant mesothelial proliferation. Mesothelial cysts can occur in any area covered by mesothelium, such as the mesentery, omentum, mediastinum, round ligament of the uterus, and adnexa, with a primary involvement of the pelvic and abdominal peritoneum (13). Ultrasonography, CT, and MRI lack specificity in diagnosing this condition, and definitive diagnosis relies on pathological and immunohistochemical examinations (14). Histopathological sections typically show a single layer of cuboidal epithelium, rarely stratified, without proliferation. The nuclear-to-cytoplasmic ratio is normal, and there is no nuclear atypia or mitotic activity. The cell polarity is normal. Immunohistochemical staining for mesothelial markers such as MC, CR, CK8/18, Ki-67, CK5/6, WT-1, and mesothelial cells can aid in differential diagnosis (15). Mesothelial cysts need to be differentiated from multicystic mesothelioma. According to the World Health Organization’s classification of soft tissue tumors, multicystic mesothelioma is categorized as a benign or low-grade malignant mesothelioma. Benign multicystic mesothelioma is composed of multiple cysts of varying sizes, often containing thin serous fluid. Immunohistochemical markers show expression of CK7, CK5/6, WT-1, and calretinin in mesothelial cells. In contrast, malignant multicystic mesothelioma exhibits significant cellular atypia, with a disordered growth pattern and increased mitotic figures. Immunohistochemical markers for this condition include Ki-67(+) and EMA(−) (16). Mesothelial cysts are treated with surgical resection, resulting in excellent prognosis with minimal risk of recurrence or malignancy. For low-grade malignant mesothelioma, surgery is combined with adjuvant chemotherapy and close follow-up.

The co-occurrence of HWWS and mesothelial cysts is rare, and the pathogenic mechanisms linking these two conditions require further investigation. The post-septal cavity in HWWS can lead to retrograde menstruation, which often results in pelvic endometriosis, making ovarian endometriotic cysts on the affected side the most common complication (17, 18). Pathological examination of typical endometriosis reveals endometrial glands and surrounding stroma, often accompanied by fresh or old hemorrhage, with immunohistochemical positivity for CD10. In the present case, pathological diagnosis ruled out endometriosis, confirming the diagnosis of HWWS co-occurring with a mesothelial cyst.

Analysis of the misdiagnosis in this case: (1) the important clinical history of “intermittent lower abdominal pain exacerbated during the last two menstrual periods” was overlooked. (2) Key CT findings, including “uterus didelphys, unilateral pelvic effusion, and renal agenesis,” were ignored, and no further MRI examination was conducted. (3) Two auxiliary examinations led to misdiagnosis, and the reasons are as follows: (1) renal scintigraphy misdiagnosis: firstly, renal scintigraphy has limitations, with non-intuitive images. Secondly, in patients with renal agenesis, the tracer may accumulate in other organs such as the spleen or uterus, leading to potential misdiagnosis. Therefore, we believe that enhanced CT is significantly superior to renal scintigraphy for diagnosing ectopic kidneys and renal agenesis. (2) Ultrasonography misdiagnosis: first, the disease is extremely rare, and clinicians may lack familiarity with its ultrasonographic manifestations or perform insufficiently detailed examinations. Second, the patient’s left renal area was vacant, suggesting either an ectopic kidney or renal agenesis. Mesothelial cysts appear as unilocular or multilocular anechoic cystic masses, which are difficult to distinguish from an ectopic polycystic kidney dysplasia on two-dimensional ultrasound. Finally, the patient’s ineligibility for vaginal ultrasound examination to differentiate the condition was also a contributing factor.

The current treatment for HWWS is mainly resection of the oblique vaginal septum, thereby addressing menstrual irregularities, dysmenorrhea, and abdominal pain. Clinicians should integrate postoperative follow-up for HWWS to ensure the duration and frequency of follow-up, preventing recurrence and secondary malignancies.

## Conclusion

In summary, the co-occurrence of HWWS and mesothelial cysts is clinically rare. Preoperative assessment should include a thorough medical history and physical examination, as well as a detailed review of laboratory and imaging results. A comprehensive analysis combining medical history, physical findings, and diagnostic tests is essential, especially when there is a discrepancy between test results and clinical presentation. Physicians should not rely solely on test reports and must maintain a clear understanding and heightened vigilance for differential diagnosis. In adolescent females with renal agenesis or ectopic kidneys presenting with gynecological symptoms, vigilance for HWWS is crucial, particularly in cases of acute presentations without menarche, where the possibility of genital tract malformations should be considered. Vaginal ultrasonography and MRI are effective tools for diagnosing and clinically subtyping HWWS (19). The complexity of HWWS, along with its associated complications, makes it prone to misdiagnosis and missed diagnosis. A detailed evaluation of the patient’s medical history, symptoms, and physical signs, combined with ultrasonography and pelvic MRI, is necessary to formulate the optimal treatment plan and improve patient outcomes. Mesothelial cysts are benign lesions with good prognosis. However, post-operative monitoring is essential to reduce the risk of recurrence and malignant transformation. Further research is needed to explore any potential pathogenic associations between HWWS and mesothelial cysts.

## Data Availability

The original contributions presented in the study are included in the article/supplementary material, further inquiries can be directed to the corresponding authors.
